# Supplementary material for: Have Lifestyle Habits and Psychological Well-Being Changed among Adolescents and Medical Students Due to COVID-19 Lockdown in Croatia?
Source: Nutrients. 2020 Dec 30;13(1):97. doi: 10.3390/nu13010097 (PMC7830522; doi:10.3390/nu13010097)
Supplement: Supplementary file 1 [file nutrients-13-00097-s001.pdf]

Supplemental table 1. Dietary habits of the sample before and during COVID-19 lockdown and comparison between three study groups

	**Pre-COVID-19 (2018 and 2019)**				**COVID-19 lockdown**			
	Secondary school students N=769	Domestic medical students N=413	International medical students N=144	P	Secondary school students N=324	Domestic medical students N=148	International medical students N=59	P
Breakfast frequency; N (%)				0.022*				0.217*
every day	457 (59.9)	233 (56.6)	67 (46.5)		201 (62.0)	94 (63.5)	29 (49.2)	
4-6 days/week	145 (19.0)	95 (23.1)	34 (23.6)		63 (19.4)	24 (16.2)	12 (20.3)	
0-3 days/week	161 (21.1)	84 (20.4)	43 (29.9)		60 (18.5)	30 (20.3)	18 (30.5)	
Number of main meals on working days; median (IQR)	3.0 (0.0)	3.0 (1.0)	3.0 (1.0)	0.001#	3.0 (0.0)	3.0 (1.0)	2.0 (1.0)	<0.001#
Number of main meals on non-working days; median (IQR)	3.0 (1.0)	3.0 (1.0)	3.0 (1.0)	0.019#	na	na	na	na
Number of snacks on working days; median (IQR)	2.0 (1.0)	2.0 (1.0)	2.0 (1.0)	0.020#	2.0 (2.0)	2.0 (1.0)	1.0 (1.0)	0.013#
Number of snacks on non-working days; median (IQR)	2.0 (1.0)	2.0 (2.0)	2.0 (1.0)	<0.001#	na	na	na	Na
Snacking while watching TV or studying†; N (%)				0.096*				0.048*
yes, frequently	58 (14.8)	36 (17.5)	21 (15.6)		43 (13.3)	21 (14.2)	15 (25.4)	
yes, sometimes	236 (60.2)	101 (49.0)	81 (60.0)		187 (57.7)	72 (48.6)	28 (47.5)	
no	98 (25.0)	69 (33.5)	33 (24.4)		94 (29.0)	55 (37.2)	16 (27.1)	
Sweets daily intake; N (%)	318 (41.4)	179 (43.6)	29 (20.4)	<0.001*	101 (31.2)	34 (23.0)	14 (23.7)	0.135*
Sweetened drinks daily intake; N (%)	270 (35.3)	96 (23.3)	20 (14.0)	<0.001*	75 (23.1)	25 (16.9)	13 (22.0)	0.302*
Processed meat and fish foods daily intake; N (%)	170 (22.2)	51 (12.3)	19 (13.3)	<0.001*	56 (17.3)	14 (9.5)	7 (11.9)	0.068*
**Mediterranean diet components**								
Daily fruits consumption (≥2 servings); N (%)	431 (56.0)	243 (58.8)	103 (71.5)	<0.001*	218 (67.3)	93 (62.8)	36 (61.0)	0.488*

Daily vegetables consumption (≥2 servings); N (%)	112 (14.6)	115 (27.8)	59 (41.0)	<0.001*	68 (21.0)	29 (19.6)	21 (35.6)	0.031*
Daily cereals consumption (≥2 servings); N (%)	301 (39.1)	125 (30.3)	46 (31.9)	0.032*	78 (24.1)	38 (25.7)	12 (20.3)	0.720*
Daily olive oil consumption (≥2 servings); N (%)	80 (10.4)	49 (11.9)	31 (21.5)	0.004*	35 (10.8)	17 (11.5)	11 (18.6)	0.227*
Daily milk and dairy products consumption (2 servings); N (%)	243 (31.6)	124 (30.0)	55 (38.2)	0.142*	82 (25.3)	26 (17.6)	11 (18.6)	0.132*
Daily nuts consumption (≥1 servings); N (%)	112 (14.6)	91 (22.0)	47 (32.6)	<0.001*	43 (13.3)	27 (18.2)	10 (16.9)	0.342*
Weekly fish consumption (≥2 servings); N (%)	179 (23.3)	116 (28.1)	29 (20.1)	0.058*	100 (30.9)	57 (38.5)	17 (28.8)	0.205*
Weekly white meat consumption (2 servings); N (%)	210 (27.3)	90 (21.8)	34 (23.6)	<0.001*	80 (24.7)	44 (29.7)	12 (20.3)	0.313*
Weekly red meat consumption (1 serving); N (%)	229 (29.8)	124 (30.0)	81 (56.3)	<0.001*	96 (29.6)	50 (33.8)	34 (57.6)	<0.001*
Weekly legumes consumption (≥2 servings); N (%)	345 (44.9)	270 (65.4)	92 (63.9)	<0.001*	186 (57.4)	98 (66.2)	38 (64.6)	0.157*
Weekly sweets consumption (≤2 servings); N (%)	145 (18.9)	99 (24.0)	53 (36.8)	<0.001*	93 (28.7)	55 (37.2)	14 (23.7)	0.088*
**Overall Mediterranean diet**								
MDSS score; median (IQR)	7.0 (5.0)	8.0 (6.0)	9.0 (6.0)	<0.001#	7.0 (5.0)	8.0 (5.0)	8.0 (7.0)	0.553#
MD compliant; N (%)	75 (9.8)	51 (12.3)	34 (23.6)	<0.001*	28 (8.6)	13 (8.8)	8 (13.6)	0.475*

IQR - interquartile range, MDSS – Mediterranean Diet Serving Score, MD – Mediterranean diet, na – not applicable; *chi-square test, #Kruskal-Wallis test, †data available only for year 2019 for pre-COVID study period (sample size is 733).

Supplemental table 2. Sleeping habits, physical activity, and psychological well-being characteristics of the sample before and during COVID-19 lockdown and comparison between three study groups

	**Pre-COVID-19 (2018 and 2019)**				**COVID-19 lockdown**			
	Secondary school students N=769	Domestic medical students N=413	International medical students N=144	P	Secondary school students N=324	Domestic medical students N=148	International medical students N=59	P
**Sleeping habits**								
Sleep duration on working days; median (IQR)	7.3 (1.5)	7.0 (1.5)	7.8 (1.0)	<0.001#	8.5 (1.5)	8.0 (1.2)	8.0 (1.8)	0.009#
Feeling after waking up on working days; N (%)				<0.001*				0.100*
refreshed	40 (5.2)	55 (13.3)	17 (11.8)		99 (30.6)	56 (37.8)	12 (20.3)	
somewhat tired and sleepy	431 (56.3)	275 (66.6)	98 (68.1)		189 (58.3)	82 (55.4)	39 (66.1)	
extremely tired and sleepy	295 (38.5)	83 (20.1)	29 (20.1)		36 (11.1)	10 (6.8)	8 (13.6)	
Sleep duration on non-working days; median (IQR)	9.0 (2.0)	9.0 (1.0)	8.5 (1.0)	<0.001#	na	na	na	na
Feeling after waking up on non-working days; N (%)				0.031*				na
refreshed	395 (51.8)	251 (60.9)	76 (52.8)		na	na	na	
somewhat tired and sleepy	323 (42.3)	146 (35.4)	62 (43.1)		na	na	na	
extremely tired and sleepy	45 (5.9)	15 (3.6)	6 (4.2)		na	na	na	
**Physical activity**								
Physical activity frequency† N (%)	N=388	N=205	N=67	0.001*				0.359*
weekly	236 (60.8)	108 (52.7)	51 (76.1)		216 (66.7)	89 (60.1)	37 (62.7)	
sometimes	51 (13.1)	25 (12.2)	10 (14.9)		50 (15.4)	21 (14.2)	11 (18.6)	
rarely or never	101 (26.0)	72 (35.1)	6 (9.0)		58 (17.9)	38 (25.7)	11 (18.6)	
Sitting time (h/day); median (IQR)	7.5 (4.0)	6.0 (3.3)	7.0 (5.0)	<0.001#	5.0 (5.0)	6.0 (5.0)	10.0 (3.5)	<0.001#
TV watching time (h/day); median (IQR)	1.0 (1.8)	0.5 (1.0)	0.5 (1.9)	<0.001#	1.0 (1.7)	1.0 (1.8)	1.0 (2.0)	0.344#
Computer/tablet use time	0.5 (1.0)	1.0 (1.8)	2.0 (3.0)	<0.001#	3.0 (3.5)	2.0 (2.9)	6.0 (6.5)	<0.001#

(h/day); median (IQR)								
Mobile use time (h/day); median (IQR)	3.5 (2.5)	3.0 (3.0)	2.0 (1.5)	<0.001#	4.0 (3.2)	3.0 (2.0)	2.5 (2.5)	<0.001#
Studying time (h/day); median (IQR)	2.0 (2.0)	4.0 (3.0)	4.0 (3.0)	<0.001#	3.0 (3.5)	3.5 (3.4)	6.0 (5.0)	<0.001#
**Psychological well-being**								
Happiness; median (IQR)	7.0 (3.0)	8.0 (2.0)	7.0 (2.0)	0.001#	7.0 (3.0)	7.0 (2.0)	7.0 (4.0)	0.085#
Optimistic about future; median (IQR)	7.0 (4.0)	7.0 (3.0)	8.0 (3.0)	0.176#	6.0 (3.0)	7.0 (3.0)	7.0 (3.0)	0.319#
Anxiousness; median (IQR)	4.0 (5.0)	3.0 (4.0)	5.0 (5.0)	0.002#	4.0 (5.0)	4.0 (5.0)	4.0 (5.0)	0.670#
Perceived stress score (PSS); median (IQR)	20.0 (11.0)	16.0 (9.0)	19.0 (11.0)	<0.001#	19.0 (10.0)	19.0 (10.0)	22.0 (11.0)	0.059#
Perceived stress category; N (%)				<0.001*				0.252*
low	174 (22.7)	126 (30.5)	40 (28.0)		69 (21.3)	33 (22.3)	10 (16.9)	
moderate	436 (57.0)	261 (63.2)	81 (56.6)		206 (63.6)	96 (64.9)	34 (57.6)	
high	155 (20.3)	26 (6.3)	22 (15.4)		49 (15.1)	19 (12.8)	15 (25.4)	
Quality of life; median (IQR)	8.0 (2.0)	8.0 (2.0)	8.0 (2.0)	0.225#	7.0 (3.0)	7.0 (2.0)	7.0 (4.0)	0.003#

IQR - interquartile range, na – not applicable; *chi-square test, #Kruskal-Wallis test; †data available only for year 2019 for pre-COVID study period (sample size is 66

Supplemental table 3. Correlations between lifestyle characteristics and psychological characteristics in the pre-COVID-19 sample (N=1326), data are presented as Spearman's rho, P value.

	BMI	MDSS score	Working days sleep	Non-working days sleep	TV time daily	Computer time daily	Mobile phone time daily	Study time daily	Sitting time daily	Stress score	Quality of life	Happiness	Anxiousness	Optimistic about future
Health rating	0.01, 0.627	0.07, 0.016	0.07, 0.017	0.06, 0.047	0.03, 0.357	-0.07, 0.008	-0.07, 0.018	-0.02, 0.502	-0.14, <0.001	-0.33, <0.001	0.40, <0.001	0.29, <0.001	-0.25, <0.001	0.27, <0.001
BMI		-0.03, 0.324	-0.05, 0.055	-0.07, 0.010	-0.08, 0.002	0.11, <0.001	-0.08, 0.009	-0.04, 0.204	-0.07, 0.018	-0.10, 0.001	0.02, 0.555	0.03, 0.296	-0.02, 0.535	0.05, 0.100
MDSS score			0.01, 0.811	-0.10, <0.001	-0.14, <0.001	0.04, 0.118	-0.16, <0.001	0.11, <0.001	-0.03, 0.281	-0.02, 0.470	0.14, <0.001	0.04, 0.155	0.00, 0.912	0.07, 0.019
Working days sleep				0.22, <0.001	0.07, 0.014	-0.08, 0.003	-0.06, 0.061	-0.14, <0.001	-0.08, 0.011	-0.09, 0.002	0.04, 0.318	0.08, 0.010	-0.08, 0.005	0.06, 0.033
Non-working days sleep					0.11, <0.001	-0.11, <0.001	0.05, 0.106	-0.06, 0.024	0.01, 0.957	0.03, 0.214	-0.03, 0.466	-0.02, 0.481	-0.01, 0.835	-0.01, 0.754
TV time daily						-0.04, 0.197	0.16, <0.001	-0.04, 0.145	0.05, 0.130	0.02, 0.397	0.01, 0.759	-0.01, 0.628	-0.06, 0.047	-0.01, 0.682
Computer time daily							-0.10, 0.001	0.09, 0.002	-0.01, 0.744	-0.03, 0.250	0.02, 0.607	-0.01, 0.911	0.04, 0.180	0.00, 0.952
Mobile phone time daily								-0.10, 0.001	0.15, <0.001	0.18, <0.001	-0.11, 0.010	-0.08, 0.013	0.09, 0.004	-0.07, 0.038
Study time daily									0.12, <0.001	0.06, 0.022	-0.01, 0.989	-0.05, 0.069	0.14, <0.001	-0.06, 0.035
Sitting time daily										0.17, <0.001	-0.17, <0.001	-0.15, <0.001	0.10, 0.003	-0.10, 0.002
Stress score (PSS)											-0.46, <0.001	-0.61, <0.001	0.68, <0.001	-0.48, <0.001

Quality of life	0.56, <0.001	-0.33, <0.001	0.39, <0.001
Happiness		-0.60, <0.001	0.48, <0.001
Anxiousness			-0.38, <0.001

Supplemental table 4. Correlations between lifestyle characteristics and psychological well-being characteristics in the COVID-19 lockdown sample (N=531), data are presented as Spearman's rho, P value

	**BMI**	**MDSS score**	**Sleep duration**	**TV time daily**	**Computer time daily**	**Mobile phone time daily**	**Study time daily**	**Sitting time daily**	**Stress score**	**Hardship during lockdown**	**Quality of life**	**Happiness**	**Anxiousness**	**Optimistic about future**
Health rating	-0.08, 0.057	0.12, 0.004	0.05, 0.246	0.01, 0.797	-0.14, 0.003	-0.04, 0.317	-0.07, 0.091	-0.17, <0.001	-0.27, <0.001	-0.02, 0.731	0.30, <0.001	0.22, <0.001	-0.25, <0.001	0.28, <0.001
BMI		-0.02, 0.655	0.02, 0.592	0.03, 0.530	-0.02, 0.639	-0.08, 0.086	-0.13, 0.004	-0.03, 0.447	-0.08, 0.059	-0.09, 0.044	0.04, 0.331	0.05, 0.244	-0.08, 0.059	-0.01, 0.990
MDSS score			-0.04, 0.334	-0.05, 0.233	0.04, 0.350	-0.09, 0.037	0.03, 0.497	-0.02, 0.727	-0.08, 0.083	-0.12, 0.007	0.15, 0.001	0.10, 0.018	-0.05, 0.233	0.08, 0.052
Sleep duration				0.16, <0.001	-0.14, 0.002	-0.02, 0.641	-0.22, <0.001	-0.22, <0.001	-0.10, 0.025	0.00, 0.956	0.06, 0.154	0.08, 0.056	-0.11, 0.013	0.09, 0.050
TV time daily					-0.10, 0.026	0.02, 0.659	-0.06, 0.197	0.01, 0.786	-0.01, 0.755	0.06, 0.157	0.09, 0.043	0.08, 0.083	-0.07, 0.095	0.05, 0.302
Computer time daily						0.03, 0.436	0.30, <0.001	0.38, <0.001	0.17, <0.001	0.03, 0.447	-0.22, <0.001	-0.19, <0.001	0.09, 0.048	-0.16, <0.001
Mobile phone time daily							-0.05, 0.251	-0.01, 0.758	0.16, <0.001	0.13, 0.002	-0.15, <0.001	-0.10, 0.017	0.16, <0.001	-0.06, 0.169
Study time daily								0.57, <0.001	0.21, <0.001	0.04, 0.427	-0.14, 0.001	-0.20, <0.001	0.15, 0.001	-0.13, 0.002

Sitting time daily	0.21, <0.001	-0.03, 0.566	-0.20, <0.001	-0.24, <0.001	0.13, 0.004	-0.11, 0.010
Stress score		0.18, <0.001	-0.54, <0.001	-0.62, <0.001	0.67, <0.001	-0.60, <0.001
Hardship during lockdown			-0.24, <0.001	-0.19, <0.001	0.17, <0.001	-0.16, <0.001
Quality of life				0.72, <0.001	-0.44, <0.001	0.50, <0.001
Happiness					-0.59, <0.001	0.53, <0.001
Anxiousnes s						-0.48, <0.001
